# Supplementary material for: Effects of moderate and high intensity isocaloric aerobic training upon microvascular reactivity and myocardial oxidative stress in rats
Source: PLoS One. 2020 Feb 7;15(2):e0218228. doi: 10.1371/journal.pone.0218228 (PMC7006926; doi:10.1371/journal.pone.0218228)
Supplement: Attachment — Submitted filename: Response letter 14-11-19.docx [file pone.0218228.s004.docx]

Rio de Janeiro, November 14^th^, 2019

-  Code: PONE-D-19-14652R1

- Title: “Effects of moderate and high intensity isocaloric aerobic training upon microvascular reactivity and myocardial oxidative stress in rats"

- Corresponding Author: Paulo Farinatti

- e-mail: [ptvf1964@gmail.com](mailto:ptvf1964@gmail.com)

Dear Editor,

Please find below our responses to the reviewers’ comments concerning our manuscript (PONE-D-19-14652R1), entitled “Effects of moderate and high intensity isocaloric aerobic training upon microvascular reactivity and myocardial oxidative stress in rats”. Thank you for the opportunity of resubmitting our manuscript.

We have addressed all the issues raised by the reviewers. The manuscript has been rewritten according to the reviewers’ suggestions, and an itemized, point-by-point response to each of the reviewers’ comments has been provided. Changes on the manuscript are marked in the “Revised Manuscript with Track Changes”, as per the received instructions.

Yours Sincerely,

Paulo Farinatti, PhD

University of Rio de Janeiro State

Salgado de Oliveira University

1) Please label appropriately the western blot images provided I have examined the revised paper as well as the figure in question and the raw data. The bands of fig. 4 are very blurry as they are in the original blot. As it seems, authors actually measured the protein levels of three different groups of rats following the intervention. There is no ladder or control in the original figure. The original figure has 9 columns that I am not sure what they represent since there are no labels. Each column must represent a different animal from one of the three experimental groups. Authors need to provide the original membranes from all the animals (24 in total) and label each column to let us know which column represents a specific animal of a specific group. Therefore, I invite you to provide the original membranes from all the animals (ideally, a control and ladder should also be provided).

Answer: We have sent the figures with the molecular weights and labels for each group. Below the pictures we placed the overlay of blot images with the colorimetric image showing the weight marker. We hope that this information will be enough to satisfy the Editor’s demand.

2) Please explain why the study design did not include a measurement of the dependent variables at baseline, to allow the evaluation of delta differences between pre- and post-training values in the three groups

Answer: Measurements of some outcomes at baseline were not feasible, since animals should be sacrificed. Alternatively, we could have added three more groups of rats to be sacrificed in control conditions (surgical procedures to expose cremaster muscle, and extraction of aorta and left ventricle). However, the value of this “control assessment” would be limited, since pre *vs*. post-training comparisons would not be inter-individual. The refinement of the experiment by doing so would not be questionable and the unnecessary sacrifice of animals would violate the ethical requisite of 3R's (Replacement, Reduction and Refinement) (Please refer to Bate S, Karp N. PLoS One 2014; 9: e114872). Given these ethical reasons, repeated measurements to evaluate deltas were performed only for variables which assessment was not invasive, and when surgery procedures were not necessary (e.g. VO_2_ max, body weight). In the case of variables invasively assessed, instead of performing repeated measurements we adopted an approach widely used in prior research with animals, by including only one control group (sedentary animals) that directly contrasted with both experimental groups (exercised animals) (Please refer to Johnson P, Besselsen D. ILAR Journal, 2002; 43: 202-6). In fact, this procedure is usually applied in studies investigating cardiovascular outcomes, particularly microcirculation (Please refer to Machado M et al. Exp Physiol, 2017 102: 1716–28; Boa B et al. PLoS ONE 2014, 9: e102554). Finally, we could claim that outcomes in our study have not been affected by potential confounding variables (as aging, diet, or exercise protocol). Thus, a single control group seemed to be enough to provide data corresponding to “baseline condition” (i.e. without any intervention).
